# PET Evidence of the Effect of Donepezil on Cognitive Performance in an Animal Model of Chemobrain

**DOI:** 10.1155/2016/6945415

**Published:** 2016-07-31

**Authors:** Ilhan Lim, Hye-Young Joung, A Ram Yu, Insop Shim, Jin Su Kim

**Affiliations:** ^1^Division of RI-Convergence Research, Korea Institute of Radiological and Medical Sciences, Seoul 01812, Republic of Korea; ^2^Department of Nuclear Medicine, Korea Cancer Center Hospital, Seoul 01812, Republic of Korea; ^3^Department of Science in Korean Medicine, College of Korean Medicine, Kyung Hee University, Seoul 02447, Republic of Korea

## Abstract

A considerable number of patients with breast cancer complain of cognitive impairment after chemotherapy. In this study, we showed that donepezil enhanced memory function and increased brain glucose metabolism in a rat model of cognitive impairment after chemotherapy using behavioral analysis and positron emission tomography (PET). We found that chemotherapy affected spatial learning ability, reference memory, and working memory and that donepezil improved these cognitive impairments. According to PET analysis, chemotherapy reduced glucose metabolism in the medial prefrontal cortex and hippocampus, and donepezil increased glucose metabolism in the bilateral frontal lobe, parietal lobe, and hippocampus. Reduced glucose metabolism was more prominent after treatment with doxorubicin than cyclophosphamide. Our results demonstrated the neural mechanisms for cognitive impairment after chemotherapy and show that cognition was improved after donepezil intervention using both behavioral and imaging methods. Our results suggested that donepezil can be employed clinically for the treatment of cognitive deficits after chemotherapy.

## 1. Introduction

A considerable number of patients with breast cancer complain of cognitive impairment after chemotherapy. Cognitive deficits in such patients consist of hippocampus-dependent memory and executive functions associated with the frontal lobe. Similar cognitive impairment was also shown in animal models [1–3]. Chemotherapy-associated cognitive dysfunction was referred to as chemobrain. Selamat and colleagues reported that awareness of cognitive changes was dependent on the healthcare and cultural context. To overcome chemobrain, various interventions such as cognitive training, methylphenidate, and erythropoietin have been performed [4–8]. Chemobrain was also found when patients with B-cell non-Hodgkin lymphomas were given rituximab, cyclophosphamide, doxorubicin, vincristine and prednisone, vincristine and rituximab, or vincristine and bendamustine [9]. Chiaravalloti's group reported that brain glucose metabolism in patients with Hodgkin disease was affected after diagnosis and during chemotherapy treatment [10]. Ponto's group reported that breast cancer survivors treated with chemotherapy may manifest long-term changes in brain glucose metabolism indicative of subtle frontal hypometabolism [11]. Simó's group reported a long-term decrease in gray matter and white matter volume in chemotherapy-treated patients [12].

Donepezil, a reversible acetylcholinesterase inhibitor, improves cognition for the patients with Alzheimer's disease and stroke traumatic brain injury and even for normal older adults [13–16]. However, to the best of our knowledge, there was no report on the effect of donepezil to chemobrain. In the present study, we investigated the effect of donepezil on memory function and brain glucose metabolism in a rat chemobrain model using behavioral test and positron emission tomography (PET).

## 2. Materials and Methods

### 2.1. Animal and the Experimental Design

Female Sprague-Dawley rats weighting 250–280 g each were used. The animals were allowed to acclimatize themselves for at least 7 days prior to the experimentation. The animals were housed in individual cages under light-controlled conditions (12/12 h light/dark cycle) and at 23°C room temperature. Food and water were made available. This experimental protocol was approved by an Institutional Review Committee for the Use of Human or Animal Subjects or the procedures are in compliance with at least the Declaration of Helsinki for human subjects or the National Institutes of Health Guide for Care and Use of Laboratory Animals (Publication number 85-23, revised 1985), the UK Animals Scientific Procedures Act 1986, or the European Communities Council Directive of 24 November 1986 (86/609/EEC). The rats were allowed to adapt to their environment at least 1 week before the experiments.

Chemobrain rat model was constructed using doxorubicin or cyclophosphamide because doxorubicin or cyclophosphamide was widely used in chemotherapy. Trimethyltin chloride (C3H9ClSn) (TMT) was also used as a control of cognitive dysfunction group for the comparison with chemobrain model. TMT is a potent neurotoxicant that selectively induces neuronal death in both human and animal limbic system and in particular in the hippocampal formation.

Rats were divided into six groups of ten individuals as follows: normal control group (group 1, *n* = 10), control group of cognitive dysfunction group induced by TMT (group 2, *n* = 10), cyclophosphamide-treated group (group 3, *n* = 10), doxorubicin-treated group (group 4, *n* = 10), cyclophosphamide-treated and donepezil administered group (group 5, *n* = 10), and doxorubicin-treated and donepezil administered group (group 6, *n* = 10). To generate a rat model of chemotherapy, 100 mg/kg of cyclophosphamide was intraperitoneally (IP) injected for groups 3 and 4. 4 mg/kg of doxorubicin was IP injected for groups 4 and 6 once a week for 3 weeks. For donepezil intervention groups, 5 mg/kg of donepezil was IP administrated every day for 3 weeks. 8 mg/kg of TMT was injected to induce cognitive dysfunction group for group 2.

### 2.2. Behavioral Study

The Morris water maze test was performed to evaluate spatial learning ability and reference memory [17]. The swimming pool of the Morris water maze was a circular water tank 200 cm in diameter and 35 cm deep. It was filled to a depth of 21 cm with water at 23. A platform 15 cm in diameter and 20 cm in height was placed inside the tank with its top surface being 1.5 cm below the surface of the water. The pool was surrounded by many cues that were external to the maze. A CCD camera was equipped with a personal computer for the behavioral analysis. Each rat received four daily trials. For 4 consecutive days, the rats were tested with three acquisition tests. They also received retention tests on the 5th day. For the acquisition test, the rat was allowed to search for the hidden platform for 180 s and the latency to escape onto the platform was recorded. The animals were trained to find the platform that was in a fixed position for 4 days for the acquisition test, and then, for the retention test (at the 5th day), they received a 1 min probe trial in which the platform was removed from the pool. The intertrial interval time was 1 min. The performance of the test animals in each water maze trial was assessed by a personal computer for the behavioral analysis (S-mart program, Spain). Passive avoidance test was also performed to assess explicit memory function [18].

### 2.3. FDG PET

Siemens Inveon PET was used in this study. Regional cerebral glucose metabolism in the same groups of rats was measured using F-18 fluorodeoxyglucose (FDG) PET after behavioral test. The FDG PET scan was performed to assess cerebral glucose metabolism for chemobrain and the effect of donepezil interventions compared to normal control. Before PET scanning, the rats were fasted for at least 8 h, after they were anesthetized with 2% isoflurane in 100% oxygen (Forane Solution; Choong Wae Pharma, Seoul, South Korea). During PET scanning, the temperature of rats was kept at 36°C with heating pads. Heart rates were monitored using a BioVet system (M2M Imaging Corp., USA). After injection of FDG (18.5 MBq/100 g body weight), 40 min of emission PET data was acquired. Transmission PET data were acquired for 15 min after emission PET scan. Emission list-mode data were sorted into 3D sinograms and reconstructed using 3D reprojection (3DRP) algorithms. No filter was applied. The image matrix was 256 × 256 × 159, the pixel size 0.155 × 0.155 mm^2^, and the slice thickness 0.796 mm.

Voxel-wise statistical analysis was performed to identify the regional differences between groups using SPM 8 (http://www.fil.ion.ucl.ac.uk/spm/). For SPM analysis, the brain region of interest was extracted and a study-specific rat brain template was constructed. Individual PET data were spatially normalized onto the rat brain template. Spatial normalization for individual PET was performed using affine and nonlinear transformations. The voxel size of spatially normalized images was 0.3 × 0.3 × 0.3 mm^3^. 3 mm of Gaussian smoothing kernel was applied for enhancing the signal to noise ratio. Count normalization was also performed. Two sample *t*-tests were used to identify regional differences between groups with a threshold of *P* < 0.05 (uncorrected).

## 3. Results

**Figure 1:**
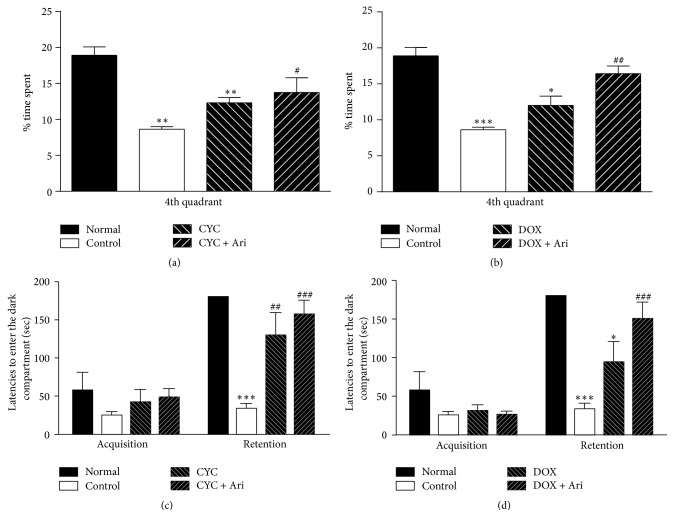
Learning and memory assessed by performance on the Morris water maze (a, b) and passive avoidance test (c, d). Time spent on the platform or 4th quadrant of the Morris water maze was recorded for the retention test (a, b). Latencies to step on the platform in the acquisition and retention trials of the passive avoidance test (c, d). Each value is represented as mean ± SEM. Statistical analysis was performed using one-way ANOVA followed by Tukey's post hoc test. ^*∗*^ *P* < 0.05, ^*∗∗*^ *P* < 0.01, and ^*∗∗∗*^ *P* < 0.001* versus *the naive group; ^#^ *P* < 0.05, ^##^ *P* < 0.01, and ^###^ *P* < 0.001* versus *the TMT group, respectively.

Figures 1(a) and 1(b) show the result of the Morris water maze test after administration of each chemotherapeutic agent, chemobrain rats needed more time to acquire information, and memory retention was impaired. After donepezil intervention these impairments were partially rescued, indicating that while chemotherapeutic agents affected spatial learning ability and reference memory, donepezil facilitated their recovery.

Figures 1(c) and 1(d) show the result of the passive avoidance test. The result shows that response times decreased after administration of each chemotherapeutic agent, and cerebral glucose metabolism was partially recovered after administration of donepezil for both the doxorubicin and cyclophosphamide induced chemobrain. This suggests that explicit working memory was impaired after chemotherapy treatment; however, donepezil intervention could improve the cognitive function. Recovery of cognitive function after administration of donepezil was more evident for doxorubicin induced chemobrain group compared to cyclophosphamide induced chemobrain group.

**Figure 2:**
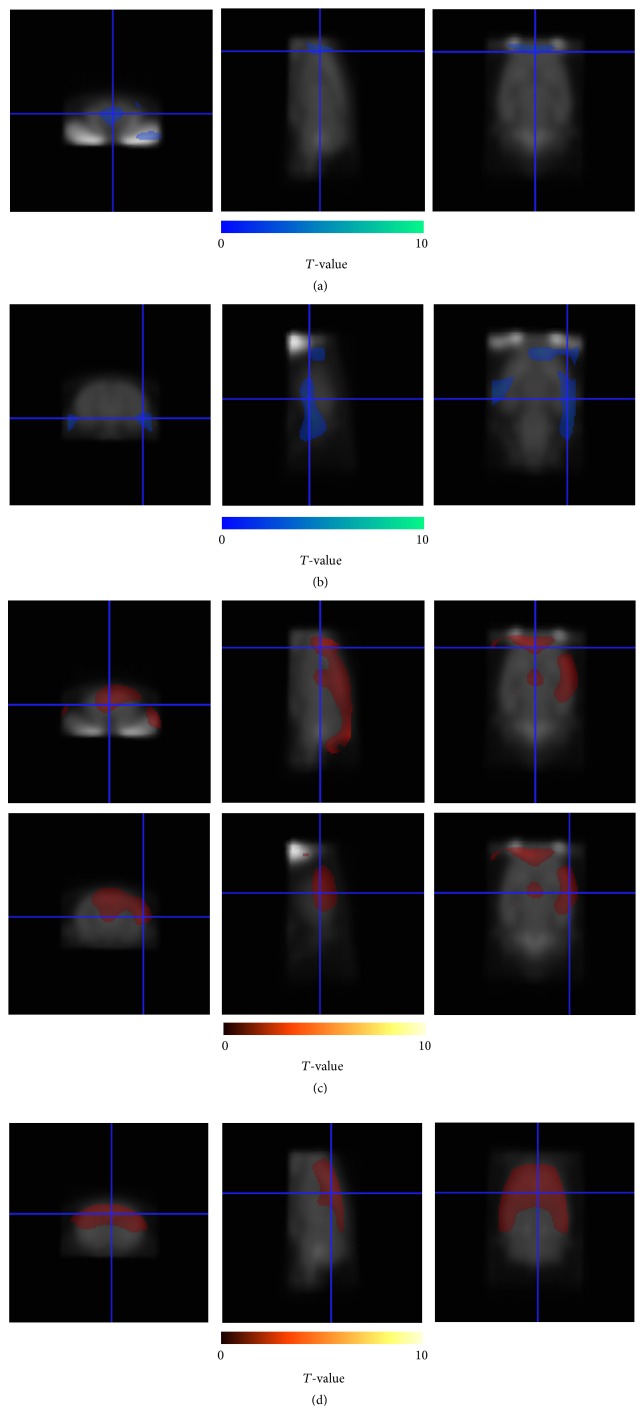
Brain regions showing decreased (blue) or increased (red) FDG brain uptake. *T*-map was overlaid on the rat brain template. Decrease in brain glucose metabolism after chemotherapy at a threshold of *P* < 0.05, uncorrected (a) in the bilateral medial prefrontal cortices for the cyclophosphamide-treated group and (b) in the left medial prefrontal cortex and bilateral hippocampi for the doxorubicin-treated group compared to normal control. Increase of brain glucose metabolism after donepezil intervention at a threshold of *P* < 0.05, uncorrected (c) in the bilateral medial prefrontal cortices, bilateral hippocampi (L > R), bilateral medial hippocampi, and bilateral parietal cortices for the cyclophosphamide-treated group and (d) in the bilateral global area of the cortices including the frontal, parietal, and temporal cortices and bilateral hippocampi for the doxorubicin-treated group, when compared with the respective chemotherapy-treated groups without donepezil intervention.

Figure 2(a) and Supplementary Figure 1a, in Supplementary Material available online at http://dx.doi.org/10.1155/2016/6945415, show the result of decrease of cerebral glucose metabolism after chemotherapy with cyclophosphamide in the region of bilateral medial prefrontal cortices (*P* < 0.05).

Figure 2(b) and Supplementary Figure 1b show that the result of cerebral glucose metabolism was decreased in the region of left medial prefrontal cortex and bilateral hippocampus after doxorubicin chemotherapy when compared with the normal untreated group (*P* < 0.05).

Figure 2(c) and Supplementary Figure 1c show the increase of glucose metabolism in the region of the bilateral medial prefrontal cortices, bilateral hippocampi (L > R), bilateral medial hippocampi, and bilateral parietal cortices after intervention with donepezil for cyclophosphamide-treated group (*P* < 0.05).

Figures 2(d) and 1(d) show the increase of glucose metabolism in the region of bilateral global area of the cortices including the bilateral frontal, bilateral parietal, bilateral temporal, and bilateral hippocampi in the doxorubicin-treated group after donepezil intervention (*P* < 0.05).

Histochemical analysis demonstrated that the number of neurons in the hippocampus and the expression of choline acetyltransferase in the hippocampus decreased after each chemotherapeutic treatment, and donepezil intervention expedited the restoration of these, although these differences were not statistically significant (Supplementary Figure 2).

## 4. Discussion

More than half (up to 75%) of the patients with cancer complain of cognitive impairment during or after treatment of their cancer. Breast cancer survivors in particular suffer from such impairment because survival after breast cancer is prolonged compared with other cancers. Longitudinal studies and meta-analyses have revealed evidence for chemotherapy-induced cognitive deficits in a subgroup of patients with breast cancer [1, 19]. The cognitive impairment ranged over diverse domains of cognition including working memory, executive function, attention, and processing speed [2]. A number of studies have used animal models of chemotherapy to understand the mechanisms underlying these cognitive changes. Cognitive impairment in patients with cancer after chemotherapy was considered significant with the increase of the survival rates of patients after cancer treatment.

Donepezil was widely used for the improvement of cognition in clinics for Alzheimer's disease, stroke, traumatic brain injury, and normal older adults. Donepezil is a reversible acetylcholinesterase inhibitor, which can facilitate the synaptic supply of acetylcholine, enhance cholinergic neural pathways, and consequently improve cognitive function [13, 14, 16, 20, 21].

In this present study, behavioral analysis revealed that chemotherapy affected spatial learning ability, reference memory, and working memory and that donepezil improved these cognitive impairments. According to PET results, chemotherapy reduced glucose metabolism in the medial prefrontal cortex and hippocampus, and donepezil increased glucose metabolism in the bilateral frontal cortices, parietal cortices, and hippocampus. Decreased glucose metabolism was more prominent after doxorubicin treatment than cyclophosphamide treatment.

In comparison with glucose metabolism change in patients after chemotherapy, results of the present study correlate with those of earlier study in that glucose metabolism decreased in the region of prefrontal cortex for rat model and glucose metabolism decreased in the region of bilateral orbitofrontal cortex in patients. It requires caution to interpret these findings because there might be significant difference between species and different chemotherapy regimens [10].

Our results revealed cognitive improvement after donepezil intervention using both behavioral and imaging methods. This suggests that donepezil could be used in clinics for the treatment of cognitive deficits after chemotherapy. The cerebral glucose metabolic responses after donepezil intervention in the present study correspond with those reported by previous studies. Using MRI, these studies reported increased activation in the bilateral prefrontal areas, inferior frontal lobes, and the left inferior parietal lobe after donepezil administration to patients with stroke [22]. They also reported selective increases of brain glucose metabolism after donepezil treatment in patients with Alzheimer's disease [23] and increased brain glucose metabolism after donepezil administration to rhesus monkeys [24].

Our results demonstrate that chemotherapy impairs cognitive function and brain glucose metabolism and that donepezil enhances these features in a rat model of chemotherapy. The present results concur with those of earlier studies, in which cyclophosphamide and/or doxorubicin affected passive avoidance learning, novel object recognition, and memory retention [25, 26], although other studies have reported no impairment after cyclophosphamide [27] or doxorubicin [28] administration. With respect to the changes in brain metabolism and structure after chemotherapy, we previously reported decreased brain glucose metabolism in the temporal lobe and cingulate gyrus after chemotherapy in patients with breast cancer [29]. Other studies have reported cerebral white matter changes [30] in the frontal and temporal lobes; gray matter changes [31] in the frontal and temporal lobes, cerebellum, and thalamus; and gray and white matter changes [32] in the prefrontal cortex, parahippocampal gyrus, and precuneus after chemotherapy. The mechanism of cognitive improvement after donepezil intervention is thought to involve modulation of cognitive function by restoring the shortage of intracerebral cholinergic neurotransmitters through inhibition of acetylcholine hydrolysis [33]. Acetylcholine plays a role as a modulator of the cortex in task-related cerebral plasticity and is essential for learning, memory, language, and attention [14, 22]. The result in our present study was well in accordance with those of a recent study [34] that reported a reduction in cognitive impairment after donepezil intervention in a mouse chemotherapy model; however, they employed a different chemotherapeutic regimen and their study was confined to behavioral examination.

The result in this present study collectively shows the effect of donepezil in a rat chemobrain model using behavioral and imaging analysis at the same time.

## 5. Conclusion

Our results suggest that donepezil may improve cognitive function and brain glucose metabolism after cognitive impairment in a rat chemotherapy model. Further clinical investigation is warranted to administer donepezil for cognitive deficits after chemotherapy.

## Supplementary Material

Supplemental figure 1 shows complete section of Figure 2. Decreased (blue) or increased (red) FDG brain uptake region was showed. T-map was overlaid on the rat brain template.
Supplemental figure 2 shows the number of choline acetyltransferase (ChAT) immunostained nuclei in the hippocampal CA1 and CA3 regions of the experimental groups. Supplemental figure 2 shows the distribution of ChAT-immunoreactive cells in the hippocampus of the different groups: normal without any treatment (A), cyclophosphamide-treated (B), cyclophosphamide-treated and donepezil administered for intervention (C), doxorubicin-treated (D) and doxorubicin-treated and donepezil administered for intervention (E). Sections were cut coronally at 30 μm.
