# Supplementary material for: PET Evidence of the Effect of Donepezil on Cognitive Performance in an Animal Model of Chemobrain
Source: Biomed Res Int. 2016 Jul 31;2016:6945415. doi: 10.1155/2016/6945415 (PMC4983340; doi:10.1155/2016/6945415)

Supplemental figure legends

Supplemental figure 1.

Complete section of Figure 2. Brain regions showing decreased (blue) or increased (red) FDG brain uptake. *T*-map was overlaid on the rat brain template.

Supplemental figure 2.

The number of choline acetyltransferase (ChAT) immunostained nuclei in the hippocampal CA1 and CA3 regions of the experimental groups. Photographs showing the distribution of ChAT-immunoreactive cells in the hippocampus of the different groups: normal without any treatment (A), cyclophosphamide-treated (B), cyclophosphamide-treated and donepezil administered for intervention (C), doxorubicin-treated (D) and doxorubicin-treated and donepezil administered for intervention (E). Sections were cut coronally at 30 μm.

Supplemental Fig 1a

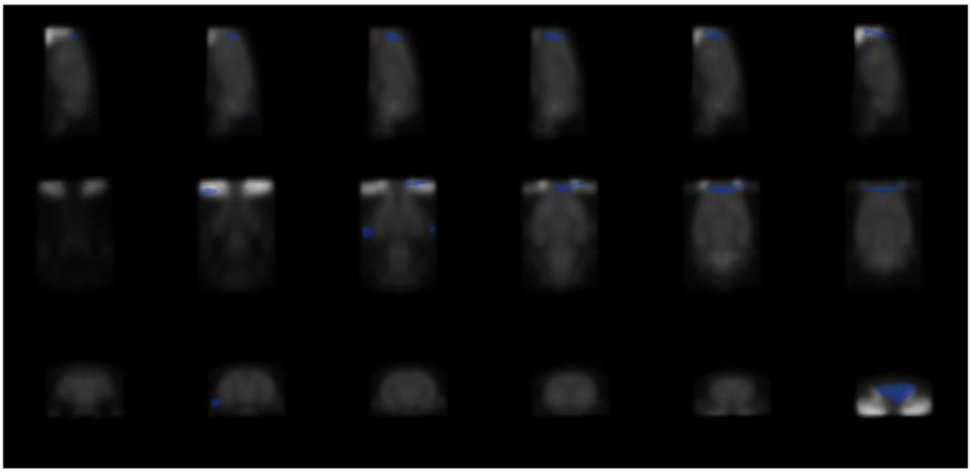

Supplemental Fig 1b

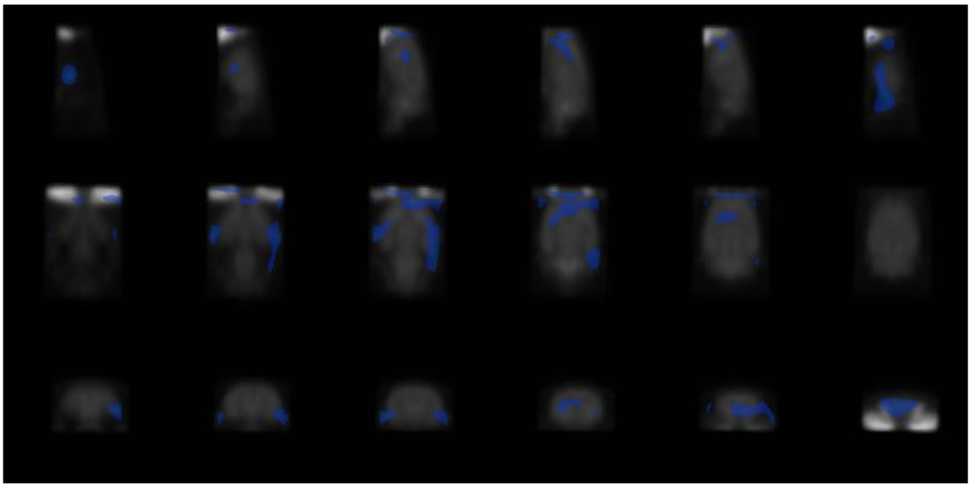

Supplemental Fig 1c

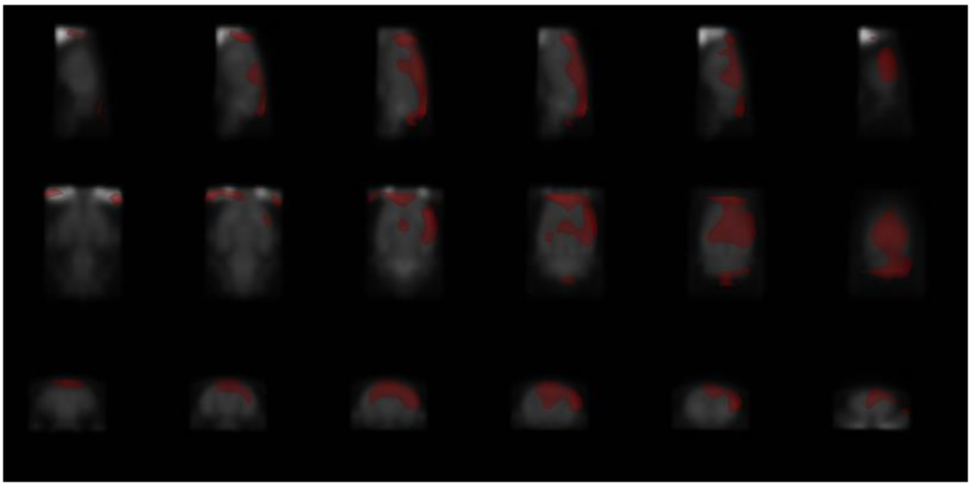

Supplemental Fig 1d

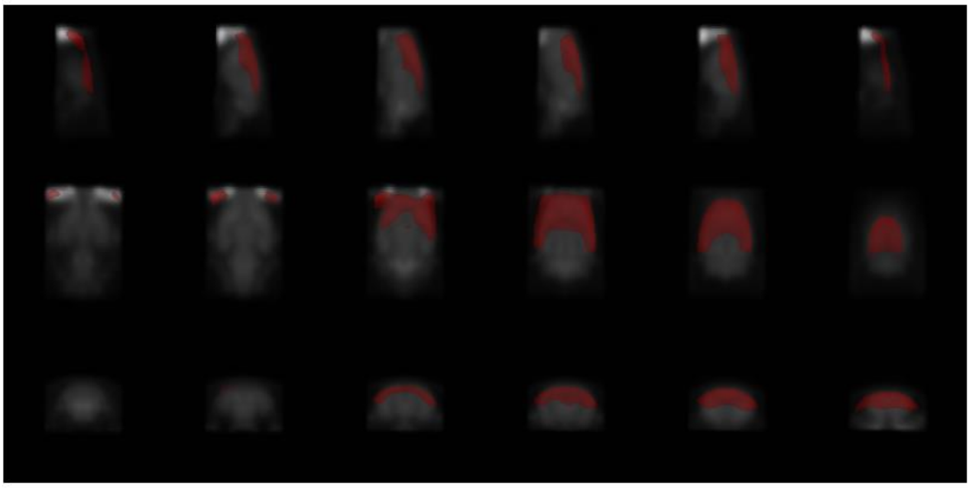

Supplemental Fig 2

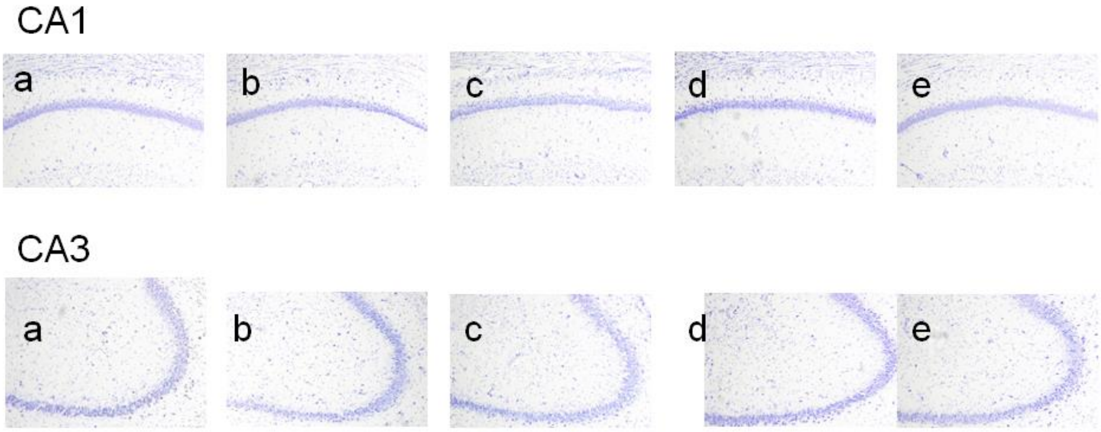

Supplement: Supplementary file 1 — Supplemental figure 1 shows complete section of Figure 2. Decreased (blue) or increased (red) FDG brain uptake region was showed. T-map was overlaid on the rat brain template. Supplemental figure 2 shows the number of choline acetyltransferase (ChAT) immunostained nuclei in the hippocampal CA1 and CA3 regions of the experimental groups. Supplemental figure 2 shows the distribution of ChAT-immunoreactive cells in the hippocampus of the different groups: normal without any treatment (A), cyclophosphamide-treated (B), cyclophosphamide-treated and donepezil administered for intervention (C), doxorubicin-treated (D) and doxorubicin-treated and donepezil administered for intervention (E). Sections were cut coronally at 30 μm. [file 6945415.f1.pdf]
